# Supplementary material for: HER2 Expression Is Predictive of Survival in Cetuximab Treated Patients with RAS Wild Type Metastatic Colorectal Cancer
Source: Cancers (Basel). 2021 Feb 5;13(4):638. doi: 10.3390/cancers13040638 (PMC7914886; doi:10.3390/cancers13040638)
Supplement: Supplementary file 1 [file cancers-13-00638-s001.pdf]

HER2 Expression Is Predictive of Survival in Cetuximab Treated Patients with RAS Wild Type Metastatic Colorectal Cancer

Said A. Khelwatty, Soozana Puvanenthiran, Sharadah Essapen, Izhar Bagwan, Alan M. Seddon and Helmout Modjtahedi

Table S1. The association between co-expression of HER family members and OS and PFS in mCRC patients treated with cetuximab as determined by Kaplan-Meier analysis and the log-rank test. P-value of ≤0.05 was considered significant.

Variables		Number of Patients	OS in Months (mean ± SE)	95% CI	*p*-value	PFS in months (mean ± SE)	95% CI	*p*-value
EGFR HER2	-ve	110	65.7 ± 3.7	58.4–72.9	0.130	58.7 ± 5.0	48.8–68.6	0.356
	+ve	34	53.0 ± 4.6	43.9–62.1		32.5 ± 4.1	24.5–40.6	
EGFR HER3	-ve	115	65.6 ± 3.7	58.4–72.8	0.136	58.9 ± 4.9	49.2–68.7	0.271
	+ve	29	53.0 ± 4.8	43.6–62.4		32.2 ± 4.3	23.8–40.7	
EGFR HER4	-ve	127	64.4 ± 3.5	57.6–71.3	0.316	55.6 ± 4.8	46.3–65.0	0.977
	+ve	17	54.8 ± 6.9	41.3–68.4		35.7 ± 5.9	24.2–47.3	
HER2 HER3	-ve	35	56.1 ± 5.9	44.6–67.6	0.147	45.9 ± 6.0	34.0–57.7	0.789
	+ve	109	65.7 ± 3.9	57.9–73.4		55.4 ± 4.9	45.8–65.0	
HER2 HER4	-ve	76	61.3 ± 4.1	53.3–69.3	0.651	42.2 ± 4.2	34.1–50.4	0.575
	+ve	68	64.5 ± 4.5	55.7–73.3		57.9 ± 6.1	45.9–69.9	
HER3 HER4	-ve	87	60.5 ± 3.7	53.2–67.7	0.535	42.2 ± 3.9	34.6–49.7	0.419
	+ve	57	65.1 ± 5.0	55.2–75.0		59.2 ± 6.7	46.1–72.2	
EGFR HER2HER3	-ve	117	65.3 ± 3.7	58.1–72.4	0.200	58.8 ± 4.9	49.1–68.5	0.287
	+ve	27	53.9 ± 5.0	44.2–63.8		32.4 ± 4.4	23.7–41.1	
EGFR HER2HER4	-ve	126	64.1 ± 3.5	57.2–71.0	0.523	56.3 ± 4.8	46.9–65.7	0.830
	+ve	18	56.8 ± 6.5	44.1–69.4		34.8 ± 5.5	24.1–45.6	
EGFR HER3HER4	-ve	129	64.2 ± 3.5	57.3–71.1	0.476	56.3 ± 4.8	46.9–65.7	0.777
	+ve	15	56.8 ± 6.46	44.1–69.4		35.6 ± 5.9	24.1–47.1	
HER2 HER3HER4	-ve	88	60.6 ± 3.7	53.3–67.8	0.560	42.5 ± 3.8	34.9–49.9	0.505
	+ve	56	64.9 ± 5.1	55.1–74.9		58.7 ± 6.7	45.2–71.6	
EGFRHER2 HER3HER4	-ve	129	64.2 ± 3.5	57.3–71.1	0.476	56.3 ± 4.8	46.9–65.7	0.777
	+ve	15	56.8 ± 6.5	44.1–69.4		35.6 ± 5.9	24.1–47.1	

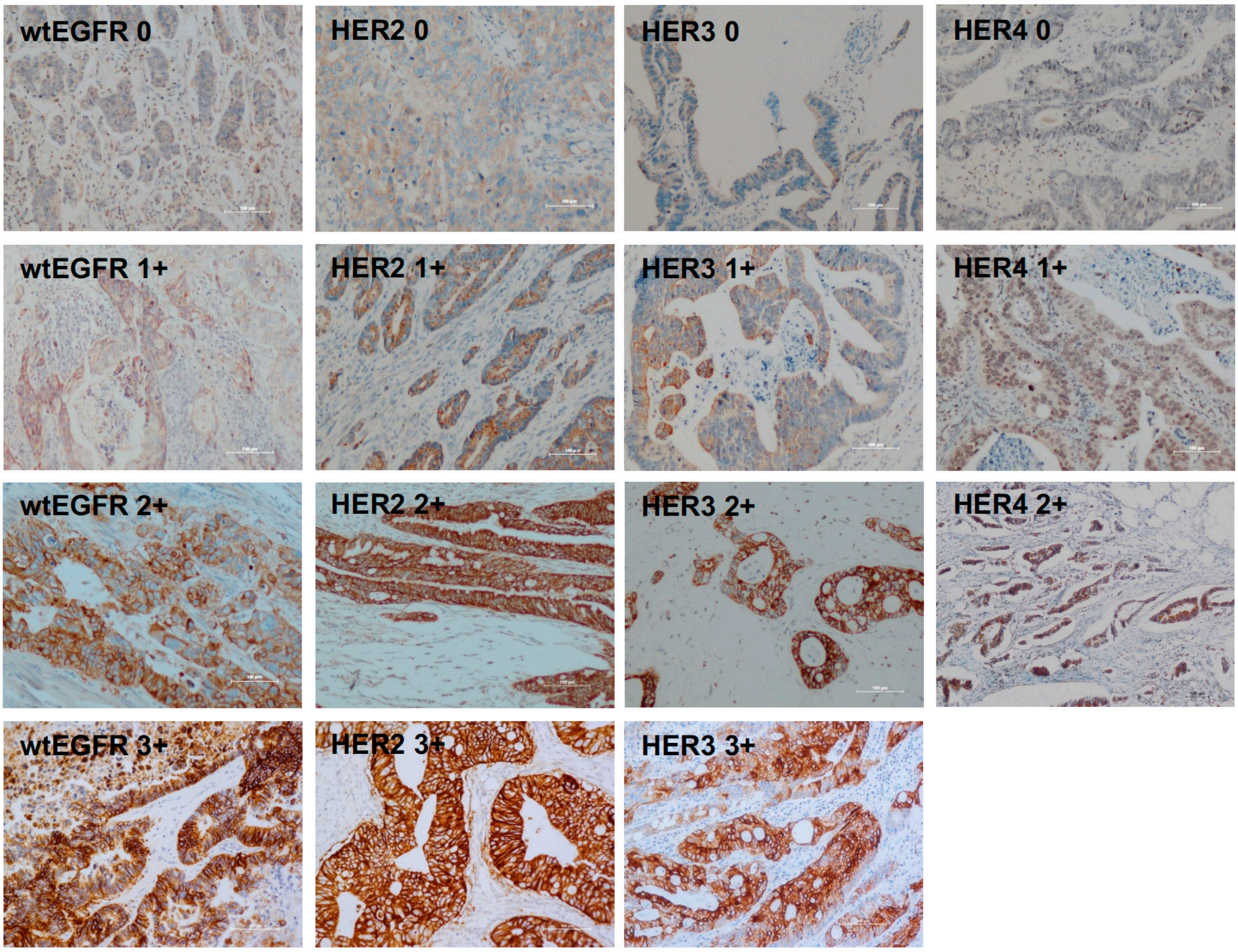

Figure S1. Immunostaining of HER family members in mCRC specimens at various intensities. Immunostaining of wtEGFR, HER2, HER3, and HER4 in formalin fixed paraffin embedded tumour sections stained immunohistochemically using the Ultra Discovery Autostainer as described previously.
